# Editorial: Different, yet strong together: the Nordic Arthroplasty Register Association (NARA)

**DOI:** 10.1080/17453674.2021.1947006

**Published:** 2021-07-07

**Authors:** Keijo Mäkelä, Nils P. Hailer

**Affiliations:** a Past NARA Chairman, Department of Orthopaedics and Traumatology, Turku University Hospital and University of Turku, Turku, Finland;; b Co-Editor/NARA Chairman, Department of Surgical Sciences—Orthopaedics, Uppsala University, Uppsala, Sweden

## Development of NARA

The Nordic Arthroplasty Register Association (NARA) was established in 2007 by hip and knee arthroplasty registry leaders from Denmark, Norway, and Sweden. Purportedly, the idea of combining national databases was presented and discussed while sitting in a bar. The 1st NARA manuscript by Havelin et al. (2009), published in Acta Orthopaedica, described differences in the demographics of patients receiving total hip arthroplasty (THA) among these 3 participating countries. The paper was well read in Finland, especially by author KM of this editorial, with disbelief and envy. It became obvious that the other Scandinavian registries were flourishing while the Finnish Arthroplasty Register (FAR) was suffering. The Finnish arthroplasty surgeons started to develop FAR in collaboration with the National Institute for Health and Welfare in Helsinki, Finland became a preliminary member at NARA meetings around 2010, and FAR data became electronic in 2014 (Finnish Arthroplasty Register 2021).

Simultaneously, the scientific work of NARA reached high standards, concerning both quality and quantity (Havelin et al. 2009, Dale et al. 2012, Mäkelä et al. 2014a, Lazarinis et al. 2017). Frequent face-to-face meetings enabled mutual confidence building between collaborators. The Scandinavians allowed NARA to be under Finnish leadership for the time period 2014–2020. “NordForsk,” a funding agency under the Nordic Council of Ministers, financially supported NARA from 2014 to 2016, and currently all participating registries support NARA financially and logistically, but there is no central funding.

All Nordic countries have similar state-funded public healthcare systems, but there are large dissimilarities between the participating countries when it comes to the practice of orthopedics, such as for example concerning fixation techniques and surgical approaches: the use of uncemented THA fixation is much more frequent in Denmark and Finland than in Norway and Sweden (Mäkelä 2014a), and the use of the posterior approach to the hip dominates in Denmark, whereas almost half of the Swedish exposures are by direct lateral approaches (Swedish Hip Arthroplasty Register 2019, Danish Hip Arthroplasty Register 2020). In total knee arthroplasty (TKA), the use of cemented fixation and of patellar resurfacing varies considerably between NARA countries (Irmola et al. 2021). These differences result in what is termed a “natural experiment” by epidemiologists, such that country-wise outcomes can be investigated (Mäkelä et al. 2014b).

Today, in June 2021, there are more than 50 scientific NARA publications, and many of them have influenced treatment practices, at least in the participating countries. For example, in 2014 a NARA paper concerning THA fixation methods in elderly patients showed that the proportion of THAs with uncemented implants had increased from 10% to 39% between 1995 and 2011, although survival for uncemented implants was much lower compared with cemented implants in patients aged ≥ 65 years (Mäkelä et al. 2014a). Gradually, cemented stems were favored in the elderly based on these and other similar data, especially in Denmark and Finland where uncemented fixation was common even in patients with hip fractures (Danish Hip Arthroplasty Register 2020, Finnish Arthroplasty Register 2021).

The role of hydroxyapatite coating in THA has been myth-busted based on NARA data. Hydroxyapatite-coated femoral stems and cups of different brands did not reduce the risk of revision due to aseptic loosening (Hailer et al. 2015, Lazarinis et al. 2017). These, together with other similar findings, suggested that hydroxyapatite coating does not render primary hip implants more stable.

Reducing dislocation rates is one of the major remaining challenges when developing modern THA further. Based on NARA data, Kreipke et al. (2019) showed that osteoarthritis patients operated on with a dual mobility cup (DMC) had a lower risk of revision due to dislocation, but a higher risk of revision caused by infection. Similarly, the use of a DMC in the treatment of patients with displaced femoral neck fractures was associated with a lower risk of revision, both for any reason and due to dislocation (Jobory et al. 2019). The total of 4,500 patients with DMC analyzed by use of the NARA database exceeded any cohort of DMC-treated hip fracture patients published previously.

NARA has increased focus towards research on TKA: In agreement with data from outside the Nordic countries (Nugent et al. 2019), Niemeläinen et al. (2020) found that both cemented and hybrid TKA had excellent 10-year survival rates in patients below 65 years, and that cemented TKA still deserves the status of gold standard in working-age patients. Another NARA paper indicated that totally uncemented TKA fixation was associated with an increased risk of revision when compared with cemented fixation in elderly TKA patients (Irmola et al. 2021).

After the success of NARA research on THA and TKA, the Nordic shoulder registries also joined the NARA collaboration. Using the NARA shoulder database, Lehtimäki et al. (2018) found that the overall mid-term risk of revision after reverse shoulder arthroplasty performed for rotator cuff tear arthropathy was low, and they identified patient factors associated with an increased risk of revision.

## NARA: the future

National datasets can provide an impressive number of observations, but conclusions are still limited when investigating rare outcomes. For instance, 90-day mortality after THA is below 0.5% (Pedersen et al. 2020) and revision due to periprosthetic joint infection affects only about 1–2% of arthroplasty patients (Dale et al. 2012). Therefore, associations of any exposure of interest with these outcomes will be muddled by large estimation uncertainty if examined on a national level alone, at least in the Nordic countries with their small populations. The NARA database provides a better opportunity to investigate rare events such as periprosthetic joint infection and mortality. The seminal paper by Dale et al. (2012) describing the increased risk of periprosthetic joint infections in all Nordic countries after the turn of the century will soon be followed by a contemporary in-depth analysis of this issue, again based on data from all NARA countries.

Designing data exchanges such as this on an international platform brings about its own difficulties in terms of jurisdiction and data governance, and administrative and regulatory problems related to sharing biometric data across borders are huge. Nonetheless, numerous challenges in modern orthopedics can only be addressed by joining forces across national borders. The already established collaboration of the Nordic countries within the NARA can provide the framework for such endeavors, even including joint ventures with other established national registries, such as the Australian Orthopaedic Association National Joint Replacement Registry (Ackerman et al. 2017) and the Dutch Arthroplasty Register (Van Steenbergen et al. 2021).

The new EU Medical Device Regulation 2017/745 (MDR) concerns all participating NARA countries. MDR represents a keystone for future medical device management in Europe and worldwide, and NARA members are strongly committed to ensuring that implants and surgical procedures are both safe and efficient. Registry-based research has provided an enormous impetus to the advancement of modern orthopedics, and all modern standards of post-market surveillance and early detection of underperforming implants are based on the continuous collection of routine data by all involved national healthcare providers. Nonetheless, registry research is observational, by definition failing to meet the rigorous standards of a scientific experiment, and the conclusions of most registry research are thus limited by the fact that causal relationships between exposures and outcomes cannot be inferred. Given these well-known limitations of observational research it is not only reasonable but even necessary to explore the potential of registries to conduct hypothesis-testing, experimental research; thus, the term “registry-nested trial” was coined (James et al. 2015).

Several Nordic national registries already provide the technical platforms needed to perform such registry-nested trials. Examples are 2 registry-based randomized controlled trials (RCT) on patients with femoral neck fractures that are nested within the Swedish Fracture Register (SFR) and the Swedish Hip Arthroplasty Register (SHAR), the “duality” and the “Hipsther” studies (Wolf et al. 2020a, 2020b). Both these studies recruit patients via a screening and randomization platform within the SFR, and the analysis of endpoints such as reoperations or implant revisions is performed via the SHAR and the National Patient Register. In Norway, a registry-nested RCT comparing the risk of periprosthetic joint infection after the use of either antibiotic-loaded or plain bone cement in the fixation of total knee arthroplasty has been initiated (Leta et al. 2021), and a Danish study comparing different regimes of perioperative antibiotics during THA surgery is in the pipeline. Future Nordic RCTs can be designed within the NARA framework, with screening and inclusion of patients performed in all 4 participating countries, and with registration of demographic and procedural details following the established routines within each participating national registry. Outcome analyses will be based either on the already established common NARA dataset, or on extended datasets, where appropriate.

In all, the NARA provides a stable framework for observational arthroplasty research, it has changed practice, at least in the participating countries, and it is a perfect platform for future experimental studies. We look forward to further contributions from this Nordic goldmine, and are open to joint ventures with other arthroplasty registries.
